# Circulating levels of inflammatory mediators in pregnant people living with HIV according to antiretroviral therapy regimen

**DOI:** 10.3389/fmicb.2023.1282291

**Published:** 2024-01-08

**Authors:** Stephanie Hindle, Sylvie Girard, Helene C. F. Cote, Deborah Money, Evelyn Mann, Isabelle Boucoiran, for the Children and Women Antiretroviral Therapy and Makers of Aging (Carma) Preg Team

**Affiliations:** ^1^Department of Pharmacology and Physiology, Université de Montréal, Montréal, QC, Canada; ^2^CHU Sainte-Justine Research Center, Montréal, QC, Canada; ^3^Department of Obstetrics and Gynecology, Université de Montréal, Montréal, QC, Canada; ^4^Department of Microbiology, Infectiology and Immunology, Université de Montréal, Montréal, QC, Canada; ^5^Department of Obstetrics and Gynecology and Department of Immunology, Mayo Clinic, Rochester, MN, United States; ^6^Department of Pathology and Laboratory Medicine, University of British Columbia, Vancouver, BC, Canada; ^7^Centre for Blood Research, University of British Columbia, Vancouver, BC, Canada; ^8^Women’s Health Research Institute, Vancouver, BC, Canada; ^9^Department of Obstetrics and Gynecology, University of British Columbia, Vancouver, BC, Canada; ^10^Oak Tree Clinic, BC Women's Hospital and Health Centre, Vancouver, BC, Canada; ^11^School of Public Health, Université de Montréal, Montréal, QC, Canada

**Keywords:** HIV, antiretroviral therapy, pregnancy, inflammation, premature birth

## Abstract

**Introduction:**

The use of antiretroviral therapy (ART) during pregnancy, particularly protease-inhibitor-based regimens (PI), has been linked to adverse outcomes including preterm delivery. As this outcome may be related to systemic inflammation, we sought to characterize inflammatory profiles of pregnant people living with HIV (PLWH) by comparing their levels of inflammatory mediators at two timepoints during pregnancy according to ART regimen, and to HIV-negative controls.

**Methods:**

Second and third trimester samples from 144 pregnant PLWH treated with ART and 24 HIV-uninfected controls were retrieved from the CARMA-PREG cohort. Peripheral plasma levels of 12 inflammatory mediators previously linked to HIV infection and/or poor pregnancy outcomes were quantified by multiplex assay: HMGB1, GM-CSF, IFNα, IFNβ, IFNγ, IL-10, IL-17, IL-1β, IL-6, TNFα, AGP, and CRP. Levels were compared by ART regimen and HIV status.

**Results:**

Adjusted analyses showed that PLWH have higher levels of AGP throughout pregnancy and lower levels of IFNγ and IL-1β during the second trimester. PI-based regimens are associated with significantly higher levels of IFNα and IL-17 during the second trimester and IFNα, CRP, HMGB1, and IFNβ during the third trimester compared to InSTI-based regimens. The PI-subgroup was associated with preterm delivery and higher HIV-1 viral load.

**Discussion:**

Our results suggest that PI-based regimens are associated with a pro-inflammatory and antiviral immunological response and a high viral load, which may be a mechanism through which PI-based regimens increase the risk of preterm delivery. Further investigations into cellular mechanisms and pro-inflammatory cascades leading to preterm delivery are necessary to support this association.

## Introduction

Despite a decreasing rate of vertical transmission of Human Immunodeficiency Virus (HIV) due to the use of Antiretroviral therapy (ART) during pregnancy, recent studies have shown that adverse pregnancy outcomes are still prevalent among people living with HIV (PLWH), especially preterm delivery (Tukei et al., 2021). In particular, in some reports, these outcomes have been associated with protease inhibitor (PI)-based regimens, although this outcome is largely variable in the literature (Favarato et al., 2018; Albert et al., 2020; Schneidman et al., 2022).

In addition, HIV status has been associated with a persistence of monocyte activation and chronic inflammation, despite the use of ART (Novelli et al., 2020). However, a study demonstrated that ART is effective in reducing levels of pro-inflammatory cytokine Interleukin-6 (IL-6) and TH2 cytokines IL-4 and IL-10, while being ineffective at increasing virally suppressed levels of TH1 cytokine Interferon-γ (IFNγ) and ineffective at reducing overly expressed Transforming Growth Factor-β (TGFβ) and Tumor necrosis factor-α (TNFα) in PLWH (Osuji et al., 2018). As pregnancy is regulated by an evolving inflammatory environment characterized by a delicate balance of pro-inflammatory and anti-inflammatory mediator release, it follows that any disruption of this balance may negatively impact pregnancy, causing adverse pregnancy outcomes, as shown previously (Nadeau-Vallee et al., 2016). Furthermore, it has been seen in some studies that PI-based regimens seem to be less effective than Integrase strand transfer inhibitors (InSTI)-based regimens in reducing viral load, as well as associated with reduced levels of progesterone, which may be associated with an increased risk of adverse birth outcomes (Papp et al., 2015, 2016; Jacobson and Ogbuagu, 2018).

To date, the pathophysiology behind the potential association between negative pregnancy outcomes and PI-based regimens in the context of HIV infection is unclear. For this reason, we aim to characterize the systemic inflammatory profile of pregnant PLWH at two time points during pregnancy using a set of 12 inflammatory mediators known to be associated with HIV-related inflammation and/or adverse pregnancy outcomes. These include pro-inflammatory mediators such as T-helper type 1 cytokine: (IFNγ); inflammation-associated cytokines: IL-1β, IL-6, IL-17, TNFα, and Granulocyte macrophage colony stimulating factor (GM-CSF) which is also an important cell differentiation modulator during pregnancy; Danger associated molecular pattern (DAMP): High mobility group box 1(HMGB1); the immunomodulatory protein alpha-1-acid-glycoprotein (AGP), which is also an acute phase protein produced in response to systemic inflammation; the major marker for acute and chronic inflammation: C-reactive protein (CRP); anti-inflammatory cytokine IL-10, and HIV-suppressive cytokines IFNα and IFNβ (Kedzierska and Crowe, 2001; Robertson, 2007; Najat Nakishbandy and Barawi, 2014; Osuji et al., 2018; Vecchié et al., 2018; Equils et al., 2020; Pereira et al., 2020; Phoswa et al., 2020; Saito Reis et al., 2021; Vyas et al., 2021). We aimed to analyze the levels of these mediators during both the second and third trimesters according to HIV status, class of ART and pregnancy outcome, namely preterm delivery or not.

## Materials and methods

### Study population

Pregnant PLWH were prospectively enrolled as part of the CARMA-PREG cohort, a sub-study of the CARMA (Children & Women AntiRetroviral therapy and Makers of Aging) prospective cohort study. Pregnant PLWH were enrolled at their first prenatal visit between 2004 and 2021 in two Canadian centers, the British Columbia Women’s Hospital in Vancouver and the *Centre Hospitalier Universitaire (CHU)* Sainte-Justine in Montreal. The cohort also includes a control group of HIV negative pregnant women recruited at the BC Women’s Hospital.

This study was approved by the ethics committees of the *Centre Universitaire Hospitalier Sainte-Justine* and the University of British Columbia. All participants provided written consent for blood collection for use in this study.

### Selection criteria

Inclusion criteria for the CARMA-PREG cohort include being pregnant, and able to consent. Additional inclusion criteria for this study were gestational age at delivery of over 20 weeks, blood samples collected during the second and third trimester of pregnancy, singleton pregnancy, and treatment with either PI or InSTI-based ART during pregnancy (with a backbone of two nucleoside/tide reverse transcriptase inhibitors). Treatment subgroups were assigned according to the ART regimen taken the longest during pregnancy. PLWH with preterm deliveries were included as long as at least one sample collected during either the second or the third trimester was available. If women had multiple pregnancies during the study period, we preferentially included pregnancies that resulted in a preterm delivery, otherwise the most recent pregnancy was included.

### Sample collection and analysis

Whole blood samples were collected in vacuum-sealed ethylenediaminetetraacetic acid (EDTA) tubes. Tubes were centrifuged, then plasma was collected and filtered at 0.45 μm, as previously described (Arshad et al., 2018). Filtered plasma was then stored in 2 mm cryovials in a −80°C freezer until analysis.

Filtered plasma inflammatory mediator levels were quantified using human multiplex custom assays using the Luminex 200 system by Eve Technologies Corp (Alberta, Canada). Twelve inflammatory mediators were selected and analyzed. The following mediators were included: GM-CSF, IFNα, IFNγ, IL-1β, IL-6, IL-10, IL-17, and TNFα were quantified using the MILLIPLEX^®^ Human Cytokine Panel A 8-Plex Custom Assay. HMGB1 and IFNβ were quantified using the MILLIPLEX^®^ Human Cytokine Panel 4 2-Plex Custom Assay. AGP and CRP were quantified using the MILLIPLEX^®^ Human Cardiovascular Disease Panel 3 2-Plex Custom Assay. All assays were run according to protocols established by Millipore, using a calculated lower limit of detection (MinDC) determined using MILLIPLEX^®^ Analyst 5.1 software, as per Eve Technologies Corp. No MILLIPLEX® 12-Plex panel were available to run all the assays on one panel due to incompatibility between certain cytokine immunoassay protocols.

### Statistical analysis

Demographic data and data pertaining to HIV-infection were compared by ART regimen as well as to HIV-negative controls using Chi-Square, ANOVA, Kruskal-Wallis, Fischer’s, and Mann–Whitney tests as appropriate, based on normality of distribution of data and category of variable, namely categorical or scale using SPSS version 28 and GraphPad Prism version 9. The timing of ART initiation was categorized as before or after conception. Gestational age at delivery was categorized as term delivery (> = 37 weeks) versus preterm delivery (<37 weeks) with due date assessed by first trimester ultrasound, or, if not available, by expert review of date of last menstruation and second trimester ultrasound. HIV viral load detection was categorized as detectable (>50 copies/ml) and non-detectable (<50 copies/ml) at the time of the blood collection.

Unadjusted analyses were performed to compare log-transformed inflammatory mediator levels by HIV status, class of ART, and gestational age at delivery (term ≥37 weeks, or preterm <37 weeks) using Brown-Forsythe and Welch ANOVA tests.

Multivariable linear regression models with each inflammatory mediator as the outcome variable were built adjusting factors previously associated with inflammatory mediator release during pregnancy, including ethnicity, time since sample collection, and substance use during pregnancy, and within PLWH subgroups, HIV viral load at the time of sampling and Cytomegalovirus (CMV) seropositivity (Velez et al., 2008; de Jager et al., 2009; Stowe et al., 2009; Simpson et al., 2020).

Logistic regression models were built to identify inflammatory mediators predictive of preterm delivery during the second trimester.

## Results

### Study sample

Of the 384 women in the whole cohort, 144 PLWH and 22 HIV-negative participants met the inclusion/exclusion criteria, for a total of 167 samples available in the second trimester, and 164 in the third trimester. Blood sample collection occurred at a mean of 21.2 weeks (SD = 5.1) during the second trimester and at a mean of 33.6 (SD = 1.7) during the third trimester. The PLWH were all on ART at the time of sampling; they were subcategorized by class of ART, namely PI (*N* = 97) and InSTI (*N* = 47). Demographic, pregnancy-related, HIV-infection-related and sample collection data are presented in Table 1. There were significantly more Black individuals in the group of PLWH compared to the HIV-negative controls. There was significantly more SGA in the PLWH groups compared to the HIV-negative controls. There were significantly more preterm deliveries in the PI subgroup than in the InSTI subgroup and the HIV-negative controls. Substance use was significantly more common in the PI subgroup and the HIV-negative control group compared to the InSTI-subgroup. Time since sample collection was significantly longer in the HIV-negative controls group compared to both groups, and longer in the PI subgroup compared to the InSTI subgroup (Mann–Whitney test). The PI subgroup had a significantly higher median HIV viral load during the second and third trimesters and a more frequent detectable HIV viral load during the second trimester compared to the InSTI subgroup.

**Table 1:** Demographic, pregnancy, and sample collection data of study population categorized by ART subgroup as well as data pertinent to HIV infection.

	**People living with HIV**		**People uninfected by HIV** (*N* = 22)	***P*-value**
	**NRTI + PI** (*N* = 97)	**NRTI + INSTI** (*N* = 47)		
**Ethnicity** (*N*, %) **White/Caucasian** **African/Black** **Other**	30 (30.9) 37 (38.1) 30 (30.9)	6 (12.8) 38 (80.9) 3 (6.4)	19 (82.6) 0 (0) 4 (17.4)	**0.001**^**a**^
**Mother’s age at delivery** (years) (median, range)	33.7 (17.6–44.9)	32.7 (21.9–44.4)	33.6 (21.6–39.7)	0.661^b^
**Pre-Pregnancy Body Mass Index** (BMI) (median, range)	25.6 (15.0–47.0)	27.4 (13.9–45.6)	23.4 (14.9–37.7)	0.124^c^
**Small for Gestational Age (SGA)** (*N*, %)	10 (10.3)	11 (23.4)	0 (0)	**0.014**^**a**^
**GA at delivery** (weeks) (median, rage)	38.3 (25.7–41.6)	39.0 (32.9–41.0)	40.1 (35.6–42.1)	**0.001**^**c**^
**Preterm birth** (*N*, %)	24 (24.7)	2 (4.3)	2 (9.1)	**0.005**^**a**^
**Substance use** (*N*, %)	48 (49.5)	5 (11.4)	13 (56.5)	**0.001**^**a**^
**Time since sample collection** (years) (median, range)	7.5 (2.5–15.5)	3.5 (1.2–8.1)	12.2 (11.6–13.7)	**0.001**^**c**^
**ART initiation at or before conception** (weeks) (*N*, %)	78 (80.4)	35 (74.5)	N/A	0.517^d^
**HIV viral load** (copies/ml) (median, range) **2nd trimester** **3rd trimester**	39 (0–30,700) 39 (0–168,423)	19 (0–15,270) 0 (0–2,658)	N/A N/A	**0.001**^**e**^ **0.001**^**e**^
**Detectable HIV-1 viral load** (>50 copies/ml) (*N*, %) **2nd trimester** **3rd trimester**	31 (32.0) 7 (8.1)	6 (12.8) 4 (8.7)	N/A N/A	**0.015**^**d**^ 1.000
**CD4 cell count** (per ml) (median, range) **2nd trimester** **3rd trimester**	530 (56–1,330)	560 (96–1,378)	N/A	0.978^e^
**Progesterone intake** (*N*, %)	4 (6.8)	3 (7.0)	N/A	1.000^d^
**Positive CMV status** (*N*, %)	82 (92.1)	46 (100.0)	N/A	0.095^d^

NRTI, nucleoside reverse transcriptase inhibitors; PI, protease inhibitors; InSTI, Integrase strand-transfer inhibitors. ^a^Chi-Square. ^b^ANOVA. ^c^Kruskal–Wallis. ^d^Fischer’s exact test. ^e^Mann–Whitney. Normality of distribution was tested using Shapiro–Wilk test, as necessary. Ethnicity and substance use were self-reported.

### HIV status

**Figure 1:**
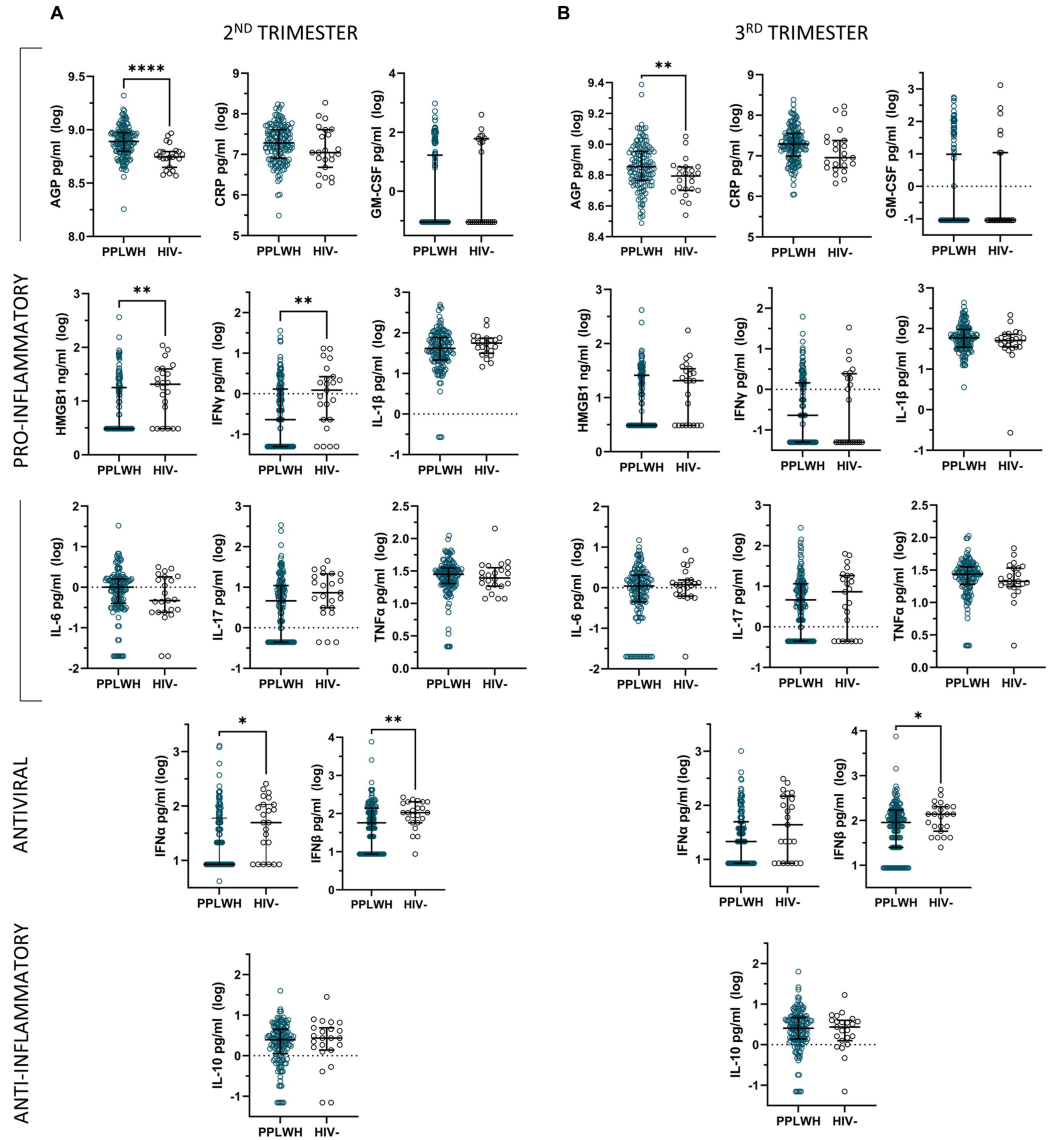
Unadjusted analyses comparing log-transformed inflammatory mediator levels by HIV status during the second **(A)** and third **(B)** trimesters of pregnancy, represented by median and interquartile range.

Unadjusted analyses comparing inflammatory mediator levels by HIV status during the second and third trimesters of pregnancy revealed significantly higher levels of AGP as well as significantly lower levels of IFNβ at both timepoints among PLWH compared to HIV negative controls (Figure 1). Furthermore, HMGB1, IFNγ, and IFNα levels were significantly lower in the PLWH group compared to the HIV negative controls during the second trimester. No other significant differences were found for the unadjusted analyses.

Multivariable linear regression models adjusting for ethnicity, time since sample collection, and substance use only maintain the significant associations for AGP and IFNγ, as well as reveal significant lower levels of IL-1β in the PLWH group during the second trimester (Supplementary Table S1).

### Antiretroviral therapy: protease inhibitors versus integrase strand transfer inhibitor

**Figure 2:**
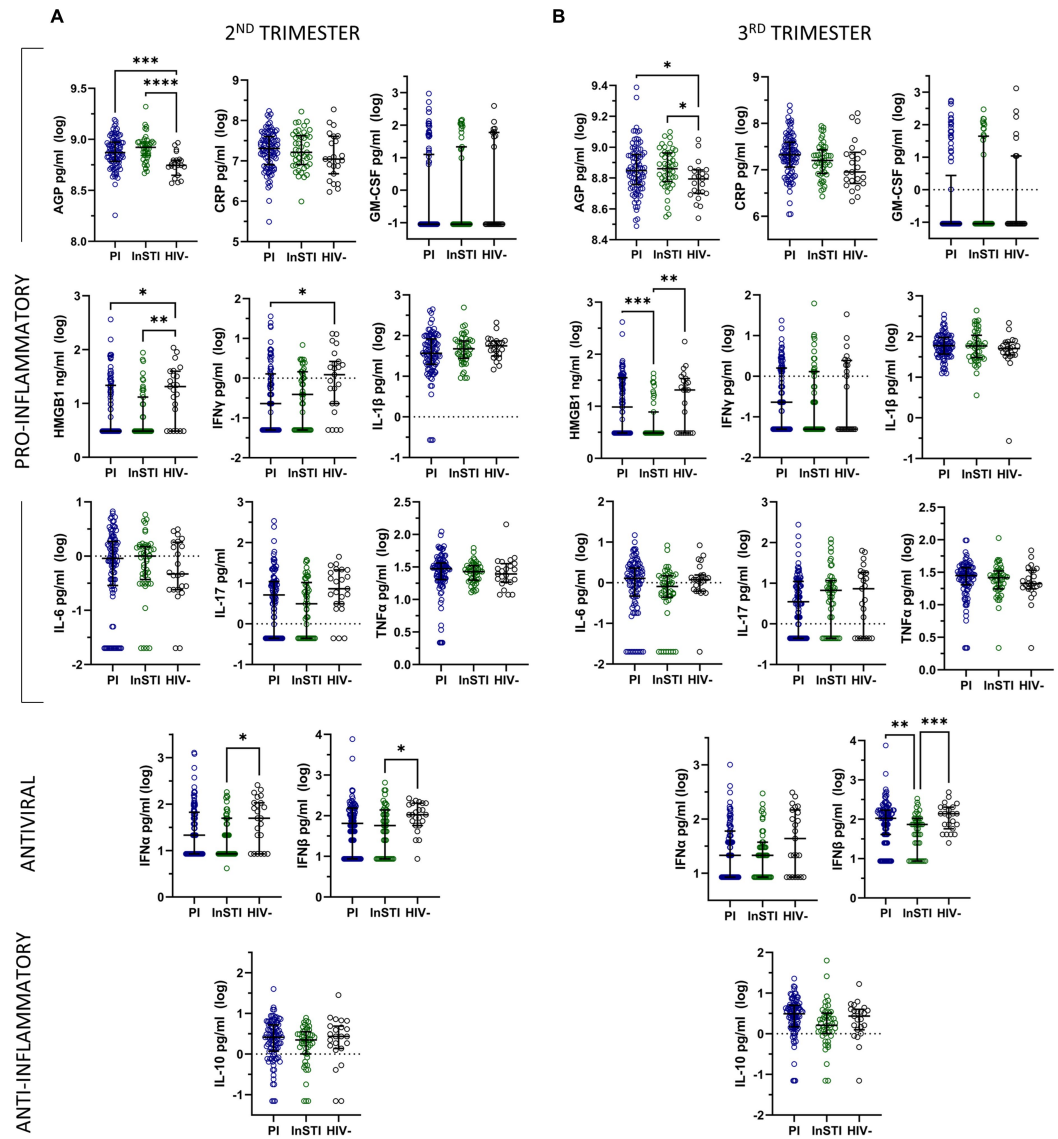
Unadjusted analyses comparing log-transformed inflammatory mediator levels by subgroup of antiretrovirals and to HIV-negative controls during the second **(A)** and third **(B)** trimesters of pregnancy, represented by median and interquartile range. (PI: protease inhibitor; InSTI: integrase strand-treansfer inhibitor).

In unadjusted analyses comparing ART subgroups to HIV-negative controls, we found that the level of AGP was significantly higher in both PI and InSTI subgroups compared to controls at both the second and the third trimester (Figure 2). During the second trimester, the level of HMGB1 was significantly higher in the control group compared to both PLWH subgroups, the level of IFNγ was significantly lower in the PI group compared to controls and the levels of IFNα and IFNβ were significantly lower in the InSTI subgroup compared to controls. During the third trimester, the levels of HMGB1 and IFNβ were significantly lower in the InSTI subgroup compared to controls and the PI subgroup.

Multivariable linear regression models adjusting for ethnicity, time since sample collection, and substance use reveal that during the second trimester, both subgroups have significantly lower levels of IFNγ and IL-1β, the InSTI subgroup has significantly lower levels of IFNα and IL-17 and the PI subgroup has significantly higher levels of AGP compared to controls. During the third trimester, both subgroups have significantly lower levels of IFNα than controls, the PI subgroup has significantly higher levels of AGP, and the InSTI subgroup has significantly lower levels of IFNβ compared to controls (Supplementary Table S2).

Multivariable linear regression models adjusting for ethnicity, time since sample collection, substance use and HIV viral load comparing both PLWH subgroups reveal that the PI subgroup had significantly higher levels of IFNα and IL-17 than the InSTI subgroup during the second trimester. During the third trimester, levels of IFNα, CRP, HMGB1, and IFNβ were significantly higher in the PI subgroup compared to the InSTI subgroup (Table 2).

**Table 2:** Parameter estimates in a linear regression model comparing log-transformed inflammatory mediator levels during the second and third trimesters of pregnancy by ART subgroup, namely PI-based regimens vs. InSTI-based regimens with the latter being the reference group, while adjusting for ethnicity, time since sample collection and substance use, as well as HIV viral load at the time of sample collection.

**Inflammatory mediator**		**2nd trimester**			**3rd trimester**		
		**Coeff. (B)**	***P*-value**	**95% C.I.**	**Coeff. (B)**	***P*-value**	**95% C.I.**
Pro-inflammatory	AGP	−0.008	0.815	−0.076 – 0.060	0.025	0.468	−0.042 – 0.091
	CRP	0.108	0.299	−0.097 – 0.313	0.228	0.019	0.038–0.418
	GM-CSF	−0.216	0.493	−0.839 – 0.406	−0.145	0.646	−0.767 – 0.478
	HMGB1	0.141	0.245	−0.098 – 0.381	0.286	0.014	0.058–0.514
	IFNγ	0.339	0.056	−0.009 – 0.687	0.093	0.633	−0.292 – 0.478
	IL-1β	0.102	0.355	−0.116 – 0.319	0.137	0.078	−0.016 – 0.289
	IL-6	−0.100	0.555	−0.435 – 0.235	0.153	0.387	−0.195 – 0.500
	IL-17	0.423	0.009	0.106–0.739	0.004	0.981	−0.322 – 0.329
	TNFα	0.022	0.751	−0.117 – 0.162	−0.022	0.747	−0.160 – 0.115
Antiviral	IFNα	0.294	0.013	0.064–0.524	0.213	0.047	0.003–0.423
	IFNβ	0.124	0.373	−0.151 – 0.399	0.333	0.008	0.087–0.578
Anti-inflammatory	IL-10	0.151	0.222	−0.093 – 0.395	0.111	0.350	−0.124 – 0.347

### Preterm delivery

**Figure 3:**
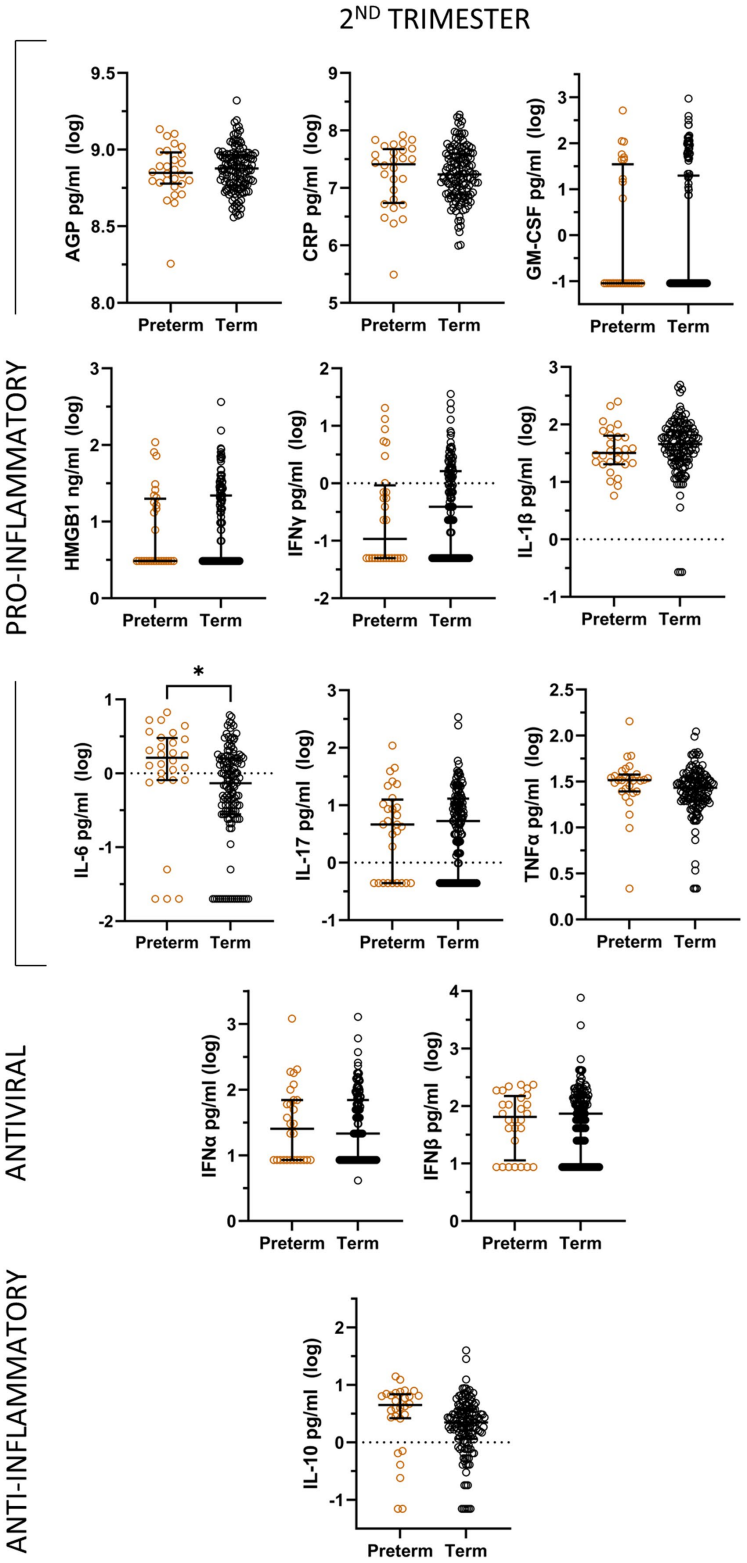
Unadjusted analyses comparing log-transformed inflammatory mediator levels by pregnancy outcome (term and preterm) during the second trimester, represented by median and interquartile range.

In unadjusted analyses, preterm delivery was associated with significantly higher levels of IL-6 during the second trimester compared to term delivery (Figure 3), although the significance of this relationship is not maintained in multivariable analyses. In logistic linear regression models, none of the second trimester levels of the inflammatory mediators were significantly associated with preterm delivery.There were not enough available samples in the third trimester for cases of preterm delivery to conduct any analyses of this timepoint.

## Discussion

### Impact of HIV status

In this study, we analyzed the levels of 12 inflammatory mediators with previously documented associations with HIV status and/or pregnancy complications. We observed significantly different levels of several pro-inflammatory mediators during pregnancy, namely AGP, IFNγ, and IL-1β, in the PLWH group compared to the HIV-negative control group (Supplementary Table S1). We demonstrate that the pro-inflammatory mediator AGP, which is involved in the regulation of every event related to inflammation, including modulation of white blood cells during acute phase inflammation, is significantly higher in the PLWH group compared to the control group at the second and the third trimester of pregnancy (Fournier et al., 2000; Ceciliani and Lecchi, 2019). This corresponds with a recent WHO/CDC expert consultation suggesting AGP as the preferred indicator for the presence of chronic infection (Rawat et al., 2009).

Moreover, multivariable analyses showed lower levels of other proinflammatory markers, IL-1β and IFNγ, during the second trimester among PLWH compared to HIV-negative controls but not in the third trimester. While peripheral levels of IL-1β have been shown to decrease during pregnancy (Denney et al., 2011; Ferguson et al., 2014), for PLWH, it increases during pregnancy, as previously demonstrated by a study including 56 South African pregnant PLWH (Akoto et al., 2021). Our results are coherent with this trend as levels of IL-1β were significantly lower during the second trimester in PLWH, while reaching similar levels as HIV negative controls in the third trimester. IFNγ levels showed a similar trend.

Furthermore, recent reports have observed that IFNγ increases IL-1β production in a time-dependent manner (Masters et al., 2010). This further supports the validity of the concurrent release of these two pro-inflammatory cytokines, although it is unclear as to why these cytokines are significantly lower in the PLWH group while the levels of AGP are significantly higher in the PLWH group compared to the HIV-negative control group.

### Impact of class of ART

Multivariable analyses comparing ART regimens show that the PI subgroup is associated with significantly higher levels of Th17 mediated cytokine IL-17 during the second trimester and antiviral cytokine IFNα at both the second and the third trimester. IL-17 is a pro-inflammatory cytokine which has been shown to promote maintenance of HIV-1 replication and activate neutrophils against infection, and has been shown to be significantly higher in PLWH compared to controls during pregnancy (Maek et al., 2007; Campillo-Gimenez et al., 2014). However, we showed that only the PI subgroup has significantly higher levels of IL-17 during the second trimester of pregnancy compared to HIV negative controls, suggesting increased inflammation in this subgroup specifically. This has been found previously, in a study which included almost only PI-based regimens (Akoto et al., 2021). This points to a dysregulation of the anti-inflammatory environment normally needed at this stage of a healthy pregnancy (Förger and Villiger, 2020).

IFNα has antiviral and immunomodulatory effects in HIV infection also known to induce inflammatory mechanisms and promote maturation of antigen presenting cells (Li et al., 2017). It was shown that the significant elevation of type I interferon in the peripheral blood of untreated, chronically HIV-1 infected people was identified as IFNα (Hardy et al., 2013). Given that the PI subgroup was also associated with a higher incidence of preterm delivery, this may suggest that the elevation of IFNα we observed in the PI subgroup may correspond to increased inflammation and immune dysregulation as compared to the InSTI subgroup.

During the third trimester, the PI subgroup is also associated with significantly higher levels of CRP, an acute pro-inflammatory mediator, HMGB1, a danger-associated molecular pattern (DAMP) and IFNβ, an antiviral cytokine that works to inhibit HIV-1 replication. Although these are not the typical markers associated with preterm delivery found in the literature, it is possible that this increase in inflammation midway through the third trimester may contribute to the significantly higher incidence of preterm delivery observed in the PI subgroup, as labour could be initiated by an upregulation of peripheral pro-inflammatory markers (Norman et al., 2007; Pandey et al., 2017). Indeed, it has been found that high levels of HMGB1 due to infection-mediated stimuli is associated with a higher risk of adverse pregnancy outcomes, such as PPROM and preterm birth (Romero et al., 2011; Plazyo et al., 2016; Saito Reis et al., 2021). Given that PPROM is responsible for 40% of all spontaneous preterm delivery, this suggests that increased HMGB1 levels in the PI-subgroup could be linked to the increased risk of preterm delivery in this subgroup (Jena et al., 2022).

### Preterm delivery

A meta-analysis as well as cohort studies have found that exposure to PI-based regimens compared to other regimens is significantly associated with preterm delivery (Mesfin et al., 2016; Schneidman et al., 2023). Additionally, induction of pro-inflammatory cytokine release has been linked to preterm delivery, although the timing of this release seems to be an important factor (Curry et al., 2007). In fact, it has been suggested that timing of inflammation during pregnancy could be a large determinant in predicting adverse outcomes (Curry et al., 2007). It was found that mid-pregnancy levels of pro-inflammatory cytokines were not significantly associated with any adverse outcomes, although they note that inflammation occurring at the fetal-placental interface is not always reflected in maternal levels of cytokines.

Increased levels of IL-6 in the second trimester are associated with preterm delivery in unadjusted analyses. This result is coherent with several other studies which have found the same association between pro-inflammatory cytokines such as IL-6 and preterm delivery in HIV negative pregnant women (Prins et al., 2012; Prairie et al., 2021).

Given that increased levels of pro-inflammatory cytokines as well as significantly higher incidence of preterm delivery are seen only in the PI subgroup of PLWH, the results of the present study support a causal association between this ART regimen and preterm delivery mediated by increased systemic inflammation. Although the suboptimal viral load control displayed in the PI subgroup of PLWH can contribute to a higher incidence of preterm delivery (Albert et al., 2020), this multivariable analysis adjusted for viral load, thereby eliminating this as a causal factor, and highlighting the impact of systemic inflammation as evidenced by the significantly higher levels of pro-inflammatory mediators in the PI subgroup.

These results suggest that InSTI-based regimens may be more effective in mitigating pro-inflammatory cell release than PI-based regimens, just as they are more effective in controlling viral load (Bastard et al., 2012; Lee et al., 2014; Jacobson and Ogbuagu, 2018; Mlambo et al., 2019). This may thereby reduce the incidence of preterm deliveries within this ART subgroup in the context of HIV chronic inflammation. It is important to note that HIV viral load was significantly higher in the PI subgroup compared to the InSTI subgroup. However, given that the association between significantly higher levels of pro-inflammatory mediator release and the PI subgroup is maintained even when controlling for viral load, the PI subgroup may induce higher levels of inflammation when compared to InSTI-based regimens through its mechanism or other intrinsic properties. These properties must be further investigated to elucidate the causal factor for this increased inflammation associated with this ART regimen.

### Study limitations

Selection of antiretroviral therapy was time dependent and somewhat related to the clinical status of the patient. This presents a potential bias in the antiretroviral based analyses. In addition, adherence to ART was not assessed directly, although it could affect the impact of ART on inflammatory mediators. Because of this, it is impossible to attribute sole causality of increased viral load in the PI subgroup to the ART class itself. In addition, it is possible that PI resistance may be partially responsible for the elevated viral load in the PI subgroup (Weber et al., 2015).

Time since sample collection is a major confounder, which was taken into account in adjusted models. However, the extent of the degradation of the samples over time and whether this is a linear or exponential effect, is unknown. Furthermore, considering each trimester as a distinct timepoint is a limitation as there is a shift in inflammatory mediator release at the end of the third trimester, especially at the time of delivery. The timing of this shift being dependent on individual characteristics, the exact timing of this shift could not be accounted for. Although this is the only study to our knowledge that compares this set of inflammatory mediators by class of ART while also adjusting for confounding variables, the sample size is relatively small, and limits the power of the analyses. Finally, inflammatory-related complications of pregnancy, such as preeclampsia, were included in the cohort, but our analyses could not evaluate the causal pathway between ART, systemic inflammation, and these complications.

It is also of note that as per the Canadian Guidelines regarding the treatment of HIV during pregnancy, all HIV infections are to be treated with antiretroviral therapy, and thus there is no HIV-infected untreated group in the present study (Transmission P.O.T.O.H.D.P.A.P.O.P, 2023).

## Conclusion and perspectives

In summary, our study presents a characterization of the systemic inflammatory mediator profile of PLWH. We observed a significantly higher viral load and levels of pro-inflammatory markers throughout pregnancy and a spike in antiviral cytokines during the third trimester associated with the PI subgroup of PLWH. These results paired with a significantly higher incidence of preterm delivery within the PI subgroup would suggest a possible causal pathway for preterm delivery. However, more investigations into the mechanisms behind this sequence of pro-inflammatory cytokine release are necessary to suggest this hypothesis as a possible explanation for the increased risk of preterm delivery associated with PI-based regimens.

## Data availbility statement

The raw data supporting the conclusions of this article will be made available by the authors, without undue reservation.

## Ethics statement

The studies involving humans were approved by Centre Universitaire Hospitalier Sainte-Justine and University of British Columbia. The studies were conducted in accordance with the local legislation and institutional requirements. The participants provided their written informed consent to participate in this study.

## Author contributions

SH: Conceptualization, Data curation, Formal analysis, Methodology, Writing – original draft, Writing – review & editing. SG: Conceptualization, Investigation, Methodology, Resources, Supervision, Validation, Writing – review & editing. HC: Conceptualization, Funding acquisition, Resources, Writing – review & editing. DM: Conceptualization, Funding acquisition, Resources, Writing – review & editing. EM: Conceptualization, Funding acquisition, Resources, Writing – review & editing. IB: Conceptualization, Funding acquisition, Investigation, Methodology, Resources, Supervision, Validation, Writing – review & editing.
