# Supplementary material for: Circulating levels of inflammatory mediators in pregnant people living with HIV according to antiretroviral therapy regimen
Source: Front Microbiol. 2024 Jan 8;14:1282291. doi: 10.3389/fmicb.2023.1282291 (PMC10801078; doi:10.3389/fmicb.2023.1282291)
Supplement: Supplementary file 1 [file Table_1.pdf]

Supp. Table 1: Parameter estimates in a multivariable linear regression model comparing log-transformed inflammatory mediator levels by HIV status during the second and third trimesters of pregnancy adjusting for ethnicity, time since sample collection, and substance use.

Inflammatory Mediator		2nd trimester			3rd trimester		
		**Coeff. (B)**	**P-value**	**95% C.I.**	**Coeff. (B)**	**P-value**	**95% C. I.**
**Pro-inflammatory**	**AGP**	0.094	**0.014**	0.019 – 0.169	0.091	**0.016**	0.018 – 0.165
	CRP	0.046	0.734	-0.219 – 0.310	0.147	0.220	-0.089 – 0.382
	GM-CSF	-0.011	0.977	-0.745 – 0.724	-0.046	0.898	-0.750 – 0.659
	HMGB1	-0.274	0.052	-0.551 – 0.003	0.016	0.905	-0.255 – 0.288
	**IFNγ**	-0.595	**0.005**	-1.009 – -0.181	-0.044	0.842	-0.479 – 0.391
	**IL-1β**	-0.327	**0.008**	-0.566 – -0.087	0.056	0.568	-0.137 – 0.248
	IL-6	0.020	0.917	-0.355 – 0.394	-0.133	0.479	-0.502 – 0.236
	IL-17	-0.271	0.157	-0.647 – 0.105	-0.299	0.111	-0.666 – 0.069
	TNFα	-0.033	0.678	-0.189 – 0.123	0.054	0.482	-0.097 – 0.205
**Antiviral**	IFNα	-0.244	0.091	-0.528 – 0.040	-0.234	0.085	-0.501 – 0.033
	IFNβ	-0.179	0.272	-0.500 – 0.142	-0.132	0.357	-0.415 – 0.150
**Anti-inflammatory**	IL-10	-0.035	0.810	-0.325 – 0.254	0.154	0.242	-0.105 – 0.412
