# Supplementary material for: Circulating levels of inflammatory mediators in pregnant people living with HIV according to antiretroviral therapy regimen
Source: Front Microbiol. 2024 Jan 8;14:1282291. doi: 10.3389/fmicb.2023.1282291 (PMC10801078; doi:10.3389/fmicb.2023.1282291)
Supplement: Supplementary file 2 [file Table_2.pdf]

Supp. Table 2: Parameter estimates in a linear regression model comparing log-transformed inflammatory mediator levels of ART subgroups to HIV-negative controls during the second and third trimesters of pregnancy adjusting for ethnicity, time since sample collection, and substance use.

Inflammatory Mediator			2nd trimester			3rd trimester		
			Coeff. (B)	P-value	95% C.I.	Coeff. (B)	P-value	95% C. I.
Pro-inflammatory	AGP	PI	0.095	**0.015**	0.019 – 0.171	0.082	**0.029**	0.008 – 0.156
		InSTI	0.094	0.063	-0.005 – 0.194	0.065	0.191	-0.033 – 0.162
	CRP	PI	0.046	0.732	-0.220 – 0.313	0.126	0.288	-0.107 – 0.360
		InSTI	-0.072	0.685	-0.421 – 0.277	-0.107	0.492	-0.416 – 0.201
	GM-CSF	PI	-0.004	0.991	-0.745 – 0.737	-0.010	0.978	-0.722 – 0.702
		InSTI	0.241	0.624	-0.727 – 1.209	0.192	0.689	-0.754 – 1.137
	HMGB1	PI	-0.289	0.043	-0.568 – 0.010	-0.026	0.847	-0.294 – 0.241
		InSTI	-0.397	0.033	-0.761 – 0.032	-0.242	0.180	-0.597 – 0.113
	IFNγ	PI	-0.605	**0.005**	-1.021 – -0.118	-0.101	0.645	-0.534 – 0.332
		InSTI	-0.836	**0.003**	-1.379 – -0.292	-0.142	0.626	-0.717 – 0.433
	IL-1β	PI	-0.340	**0.006**	-0.581 – -0.100	0.054	0.582	-0.139 – 0.246
		InSTI	-0.458	**0.004**	-0.772 – -0.144	-0.089	0.494	-0.344 – 0.167
	IL-6	PI	0.033	0.861	-0.344 – 0.411	-0.153	0.418	-0.525 – 0.219
		InSTI	0.093	0.709	-0.400 – 0.587	-0.318	0.205	-0.812 – 0.176
	IL-17	PI	-0.292	0.124	-0.666 – 0.081	-0.318	0.093	-0.689– 0.054
		InSTI	-0.654	**0.009**	-1.141 – -0.166	-0.334	0.183	-0.828 – 0.159
	TNFα	PI	-0.034	0.668	-0.192 – 0.124	0.053	0.490	-0.099 – 0.206
		InSTI	-0.051	0.629	-0.257 – 0.156	0.075	0.469	-0.128 – 0.277
Antiviral	IFNα	PI	-0.278	0.052	-0.559 – 0.003	-0.267	**0.049**	-0.533 – -0.001
		InSTI	-0.469	**0.012**	-0.836 – -0.102	-0.403	**0.026**	-0.756 – -0.050
	IFNβ	PI	-0.218	0.177	-0.535 – 0.100	-0.174	0.219	-0.454 – 0.105
		InSTI	-0.248	0.239	-0.662 – 0.166	-0.400	**0.035**	-0.771 – 0.029
Anti-inflammatory	IL-10	PI	-0.031	0.834	-0.322 – 0.260	0.146	0.272	-0.115 – 0.407
		InSTI	-0.170	0.378	-0.550 – 0.210	0.030	0.866	-0.317 – 0.376
